# Retrospective Multicenter Analysis of Intravascular Lithotripsy Use During Calcified Left Main Coronary Artery Percutaneous Coronary Interventions

**DOI:** 10.1016/j.jscai.2023.101213

**Published:** 2023-11-10

**Authors:** Robert F. Riley, Larry E. Miller, Rhian Davies, Khaldoon Alaswad, Zaid Al-Jebaje, Darshan Doshi, Farouc A. Jaffer, Srikanth Adusumalli, Jarrod D. Frizzell, Kris Kumar, Mitul P. Patel, Ali Dakroub, Ziad A. Ali

**Affiliations:** a Overlake Medical Center and Clinics, Bellevue, Washington; b Miller Scientific, Johnson City, Tennessee; c Wellspan York Hospital, York, Pennsylvania; d Henry Ford Hospital, Detroit, Michigan; e Massachusetts General Hospital, Boston, Massachusetts; f The Christ Hospital, Cincinnati, Ohio; g VA San Diego Healthcare System, San Diego, California; h St Francis Hospital and Heart Center, Roslyn, New York; i Cardiovascular Research Foundation, New York, New York; j New York Institute of Technology, Westbury, New York

**Keywords:** calcific coronary artery disease, calcium fracture, intravascular imaging, intravascular lithotripsy, left main coronary artery, percutaneous coronary intervention

## Abstract

**Background:**

Intravascular lithotripsy (IVL) safely and effectively modifies calcified coronary lesions during percutaneous coronary interventions (PCI). Data regarding its utility in modifying calcified left main coronary artery (LMCA) disease are limited. This study aimed to evaluate short-term outcomes of IVL-assisted LMCA PCI.

**Methods:**

This retrospective multicenter all-comers study analyzed patients who underwent intravascular imaging-guided, IVL-assisted PCI for calcified LMCA disease. Clinical and procedural characteristics were obtained, including intravascular imaging measurements. Technical success was defined as successful stent deployment with <30% residual diameter stenosis. Major adverse cardiac events (MACE) was a composite of all-cause death, myocardial infarction, and target vessel revascularization evaluated immediately postprocedure and at 30-day follow-up.

**Results:**

Among 184 patients treated at 7 centers from 2019-2023, IVL-assisted LMCA PCI achieved 99.4% technical success. Calcium fracture was identified in 136/165 cases (82.4%) on post-IVL imaging. Pretreatment minimal luminal area increased significantly compared to post-PCI minimal stent area (MSA) (4.1 ± 1.3 to 9.3 ± 2.5 mm^2^, respectively; *P* < .001). There was a direct correlation between IVL balloon size and the final MSA (*P* = .002). In-hospital MACE was 4.4% and 30-day MACE was 8.8%. In multivariate logistic regression, presentation with troponin-positive myocardial infarction was the sole predictor of 30-day MACE.

**Conclusions:**

IVL-assisted PCI for calcified LMCA lesions was safe and resulted in high technical success rates, confirming its utility as an effective treatment in this challenging lesion subset.

## Highlights

• Left main percutaneous coronary intervention carries significant procedural risk, particularly when these lesions are calcified.
• There is a lack of data regarding calcium modification strategies for treating calcified left main disease.
• Intravascular lithotripsy appears to be a safe and effective mechanism to treat these lesions.
• Use of larger lithotripsy balloons may result in larger minimal stent areas.

## Central Illustration

**Figure:**
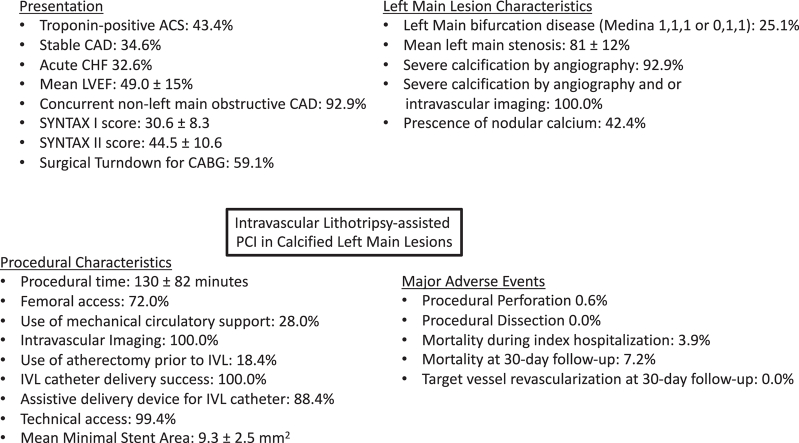

## Introduction

Patients diagnosed with obstructive left main coronary artery (LMCA) coronary artery disease (CAD) are confronted with considerable prognostic risk due to the vast myocardial territory serviced by this artery. Consequently, the American College of Cardiology and the European Society of Cardiology endorse revascularization for patients with LMCA stenosis ≥50%, irrespective of symptomatic status or related ischemic burden.^1^^,^^2^ The presence of LMCA calcification is an independent predictor of all-cause and cardiovascular death in this patient group.^3^ When calcified LMCA lesions are percutaneously treated, they are associated with higher technical failure rates, increased complication risk, and suboptimal stent expansion (with subsequent increased risk for target vessel/lesion failure) during percutaneous coronary intervention (PCI) compared to noncalcified LMCA lesions.^4^ Further, there are limited data regarding the use of traditional calcium treatment modalities such as atherectomy and specialty balloons in this lesion subset.

Intravascular lithotripsy (IVL) is increasingly used to treat calcified vascular lesions. IVL delivers controlled acoustic pressure waves that selectively fracture calcified plaques within arteries, facilitating subsequent stent expansion.^5^ While IVL has demonstrated promising results in efficiently, safely, and efficaciously treating calcified coronary lesions, data regarding its role in treating calcified LMCA lesions are limited.^6^ This study aimed to evaluate the short-term outcomes of IVL-assisted PCI in calcified LMCA lesions.

## Methods

### Study design and ethics

This retrospective multicenter study obtained Institutional Review Board approval from all participating sites. This study adheres to the Strengthening the Reporting of Observational Studies in Epidemiology (STROBE) guidelines.^7^

### Participants

The study cohort comprised consecutive patients who underwent intravascular imaging (IVI)-guided, IVL-assisted PCI for calcified LMCA lesions at 7 medical centers in the United States. The primary eligibility criteria included patients at least 18 years old with severely calcified de novo LMCA disease suitable for PCI with an indication for revascularization per the discretion of each operator, including both stable and acute coronary syndromes (ACS). The criteria for severe lesion calcification included the following: (1) angiography with fluoroscopic radio-opacities involving both sides of the arterial wall in at least 1 location and extending partially into the target lesion or (2) IVI showing ≥270° of calcium on at least 1 cross-section of the target lesion.^6^ Left main bifurcation lesions were defined as Medina 1,1,1 or 0,1,1.^8^ Patients with multivessel CAD were allowed to undergo nonleft main PCI in the same setting. There were no exclusion criteria.

### Procedure details

All patients underwent IVI with either intravascular ultrasound or optical coherence tomography pre-IVL and post-PCI; some underwent IVI after IVL treatment prior to stent implantation as well. All procedures utilized a coronary IVL catheter (Shockwave Medical) containing 2 lithotripsy emitters that generated acoustic pressure waves to selectively disrupt and fracture superficial and deep calcium. After positioning the IVL catheter across the target lesion, the IVL balloon was inflated and pulses were delivered as per prior descriptions, followed by stent placement.^6^ Patients were clinically monitored during hospitalization and for 30 days postprocedure for adverse events. Intraprocedural and posttreatment anticoagulation and antiplatelet therapy followed expert consensus guidelines.^1^

### Outcomes

Data were retrospectively collected from the electronic medical records of consecutively treated patients at each site using a standardized data collection form. The data collection included baseline patient characteristics, angiographic findings, procedural data, IVI findings, and clinical outcomes through 30 days posttreatment. Study outcomes were selected to align with those reported in the Disrupt CAD III study, using similar definitions.^6^ Technical success was defined as successful stent deployment with <30% residual stenosis. Major adverse cardiac events (MACE) included all-cause death, myocardial infarction (Q-wave and non-Q-wave), and target vessel revascularization. PCI-related myocardial infarction was defined as an increase in cardiac troponin levels to >5 × the 99th percentile of the upper reference limit during the first 48 hours following PCI plus either the following: (1) evidence of prolonged ischemia as demonstrated by prolonged chest pain; or (2) ischemic ST-segment changes or new pathological Q waves.^9^ Target vessel revascularization was defined as ischemia-driven target lesion revascularization by percutaneous or surgical methods. Serious angiographic complications were also evaluated and defined as perforation, significant (type D-F) dissection, abrupt vessel closure, slow flow, and no reflow. Procedural success was defined as technical success without MACE within the study period. IVI image assessment was performed independently at each site.

### Statistical analysis

Continuous variables were reported using the mean and standard deviation or median and interquartile range for nonnormally distributed variables. Categorical variables were reported as frequencies and percentages. Changes in the luminal area after IVL-assisted PCI were assessed using a paired samples *t* test. Univariable and multivariable logistic regression was used to identify predictors of 30-day MACE. A *P* < .05 was considered statistically significant. Statistical analyses were performed by a biostatistician using Stata v18 (StataCorp LLC).

## Results

Between January 2019 and January 2023, 184 patients from 7 centers in the United States were treated with IVL-assisted PCI for calcified obstructive LMCA CAD. Baseline patient characteristics are presented in Table 1. CAD presentation varied, with the largest proportion of patients presenting with ACS (65.4%). The majority of patients (59.1%) had been turned down for coronary artery bypass surgery prior to their PCI procedure and the mean left ventricular ejection fraction was 49% ± 15%. Mechanical circulatory support (MCS) was used in 28.0% of cases. Severe calcification was present in 90.7% of patients based on angiographic criteria, while all had significant calcification based on IVI criteria. Concurrent nonleft main obstructive CAD was noted in 92.9% of patients, and 25.1% presented with an LMCA bifurcation lesion (Table 2).

**Table 1:** Baseline patient characteristics.

Characteristic	Value
Demographics	
Male sex	69.4% (125/180)
Age, y	76 ± 0.9
Medical history	
Hypertension	91.2% (166/182)
Hyperlipidemia	86.8% (158/182)
Myocardial infarction	48.6% (88/181)
Diabetes mellitus	45.6% (83/182)
Insulin-dependent	27.5% (50/182)
Chronic kidney disease	44.8% (81/181)
Dialysis-dependent	6.1% (11/181)
CHF	42.5% (77/181)
Peripheral vascular disease	34.8% (63/181)
Chronic lung disease	23.3% (42/180)
Stroke/TIA	17.1% (31/181)
Current tobacco use	7.9% (14/177)
Procedures	
Coronary artery bypass graft	32.6% (59/181)
Pacemaker	10.5% (19/181)
ICD/CRT-D	8.8% (16/182)
Patient presentation	
LV ejection fraction, %	49 ± 15
NYHA class	
0	7.5% (11/146)
1	6.2% (9/146)
2	24.0% (35/146)
3	40.4% (59/146)
4	21.9% (32/146)
Angina class	
0	5.5% (9/165)
1	3.6% (6/165)
2	9.1% (15/165)
3	51.5% (85/165)
4	30.3% (50/165)
CAD presentation	
Troponin-positive MI	43.4% (79/182)
Stable	34.6% (63/182)
Unstable angina	22.0% (40/182)
Acute CHF presentation	32.6% (58/178)
Surgical turndown	59.1% (101/171)

The values are % (n/N) or mean ± SD.

CAD, coronary artery disease; CHF, congestive heart failure; CRT-D, cardiac resynchronization therapy-defibrillator; ICD, implantable cardioverter-defibrillator; LV, left ventricular; MI, myocardial infarction; NYHA, New York Heart Association; TIA, transient ischemic attack.

**Table 2:** Angiographic characteristics.

Characteristic	Value
Protected left main disease	26.9% (49/182)
Left main lesion location^a^	
Distal	63.4% (116/183)
Bifurcation	37.7% (69/183)
Mid	25.1% (46/183)
Ostial	8.2% (15/183)
Proximal	4.4% (8/183)
Medina classification^b^	
1,1,1	63.8% (44/69)
1,1,0	21.7% (15/69)
1,0,1	8.7% (6/69)
0,1,1	2.9% (2/69)
0,1,0	2.9% (2/69)
Left main bifurcation disease (1,1,1 or 0,1,1)	25.1% (46/183)
Reference vessel diameter, mm	3.8 ± 0.7
Stenosis, %	81 ± 12
Lesion length, mm	21 ± 15
Severe calcification by angiography	90.7% (166/183)
Calcified length, mm	17 ± 14
Concurrent nonleft main obstructive disease	92.9% (171/184)
SYNTAX score	30.6 ± 8.3
SYNTAX II score	44.5 ± 10.6

The values are % (n/N) or mean ± SD.

a Sum of percentages over 100% due to multiple lesion locations in some patients.

b Applicable to distal left main lesions.

Predilatation before IVL was performed in 83.1% of cases and atherectomy was performed before IVL in 18.4% of cases, predominantly using rotational atherectomy. IVL catheter delivery success was 100%, with a mean of 70 ± 27 IVL pulses administered during treatment. Post-IVL, prestent implantation interventions included balloon dilatation in 76.7%, with 9.7% using specialty balloons. Stent implantation was successful in 99.4% of cases. The angiographic residual diameter stenosis was 3.0% ± 5.0%, and all values were under 30%, yielding a technical success rate of 99.4%. Calcium fracture on poststent implantation IVI was identified in 82.4% of patients (Table 3). Pre-IVL minimal luminal area demonstrated a significant increase compared with post-PCI minimal stent area (MSA) (4.1 ± 1.3 to 9.3 ± 2.5 mm^2^, respectively; *P* < .001). Additionally, a larger IVL catheter diameter was associated with a greater poststent MSA. Specifically, for IVL catheter diameters of 2.5, 3.0, 3.5, and 4.0 mm, the resulting MSA were 7.6 ± 0.9, 8.4 ± 2.2, 8.5 ± 1.9, and 10.1 ± 3.2 mm, respectively (*P* = .002) (Table 4). There was also a significant difference in MSA obtained across institutions (*P* < .001).

**Table 3:** Procedural data.

Characteristic	Value
Procedure time, min	130 ± 82
Air kerma, mGy	1444 ± 1035
Contrast volume, mL	136 ± 79
Vascular access	
Femoral	72.0% (131/182)
Radial	24.7% (45/182)
Other	3.3% (6/182)
Mechanical circulatory support	28.0% (51/182)
Impella	45
Balloon pump	6
Intravascular imaging	
IVUS	90.2% (166/184)
OCT	9.8% (18/184)
Predilatation prior to IVL	83.1% (152/183)
Maximum predilatation balloon diameter, mm	2.9 ± 0.5
Atherectomy before IVL	18.4% (33/179)
Rotational	25
Orbital	5
Laser	3
IVL balloon diameter, mm	
2.5	3.4% (6/179)
3.0	32.4% (58/179)
3.5	38.5% (69/179)
4.0	25.7% (46/179)
IVL catheter delivery success	100% (184/184)
Number of IVL pulses	70 ± 27
Number of IVL catheters used for left main treatment	
1	90.7% (166/183)
2	8.2% (15/183)
3 or more	1.1% (2/183)
Assistive catheter delivery device (guide extension or buddy wire) used	88.4% (160/181)
Post-IVL balloon dilatation (prestent)	76.7% (138/180)
Stent delivery success	99.4% (180/181)
Number of stents implanted	
0	1.1% (2/183)
1	55.7% (102/183)
2	31.1% (57/183)
3 or more	12.0% (22/183)
Total stent length, mm	39±26
Poststent dilatation	96.7% (178/184)
Maximum postdilatation balloon diameter, mm	4.4 ± 0.6
Left main bifurcation stenting	33.1% (46/183)
DK mini-crush	33
Culotte	7
TAP	5
V-stent	1
Residual diameter stenosis, %	3 ± 5
<30%	100% (184/184)
Technical success	99.4% (180/181)
Hospital stay, d^a^	2 (1-4)

The values are mean ± SD or % (n/N), unless otherwise indicated.

DK, double kissing; IVL, intravascular lithotripsy; IVUS, intravascular ultrasound; OCT, optical coherence tomography; TAP, T and protrusion.

a Median (IQR).

**Table 4:** Intravascular imaging.

Characteristic	Value
Pre-IVL imaging	
Minimal luminal area, mm^2^	4.1 ± 1.3
Calcium length, mm	13.1 ± 6.6
Largest arc of calcium, °	279 ± 81
Maximal calcium thickness, mm	1.3 ± 0.5
Nodular calcium	42.4% (67/158)
Post-PCI imaging	
Minimal stent area, mm^2^	9.3 ± 2.5
Calcium fracture	82.4% (136/165)

Values are mean ± SD or % (n/N).

IVL, intravascular lithotripsy; PCI, percutaneous coronary intervention.

In-hospital MACE was 4.4%, comprising all-cause death (3.9%) and myocardial infarction (2.2%), with no target vessel revascularization (0%). Final angiographic complications were rare, including perforation (0.6%), abrupt vessel closure (0.6%), slow flow (1.1%), and no reflow (0.6%). The 30-day MACE was 8.8%, including all-cause death (7.2%) and myocardial infarction (3.3%), without target vessel revascularization (0%) (Table 5). Overall, the 30-day procedural success rate was 90.6% (164/181).

**Table 5:** Complications.

Complication	Value
Final angiographic complications	
Perforation	0.6% (1/181)
Significant (type D-F) dissection	0.0% (0/181)
Abrupt vessel closure	0.6% (1/181)
Slow flow	1.1% (2/181)
No reflow	0.6% (1/181)
Hospital MACE	4.4% (8/181)
Death	3.9% (7/181)
Cardiac	5
Noncardiac	2
MI	2.2% (4/180)
Q-wave	2
Non-Q-wave	2
TVR	0.0% (0/180)
30-day MACE	8.8% (16/181)
Death	7.2% (13/181)
Cardiac	5
Noncardiac	8
MI	3.3% (6/180)
Q-wave	2
Non-Q-wave	4
TVR	0.0% (0/180)

MACE, major adverse cardiac event; MI, myocardial infarction; TVR, target vessel revascularization.

In univariable logistic regression, presentation with a troponin-positive myocardial infarction (non-ST-segment elevation myocardial infarction or ST-segment elevation myocardial infarction), acute congestive heart failure presentation, history of congestive heart failure, and chronic lung disease were associated with increased risk for 30-day MACE. In multivariable logistic regression, presentation with a troponin-positive myocardial infarction was the sole predictor of 30-day MACE (Supplemental Table S1).

## Discussion

This study evaluated the short-term outcomes of IVI-guided, IVL-assisted PCI for calcified LMCA lesions. This represents the largest multicenter registry evaluating the use of IVL in LMCA PCI in an all-comer population. The results demonstrated high technical success rates, significant increases in luminal area post-PCI, and low rates of short-term MACE, suggesting a favorable safety profile in this patient population. These findings support IVL as a safe, effective strategy for treating calcified LMCA lesions in patients undergoing percutaneous revascularization (Central Illustration).

**Central Illustration:**
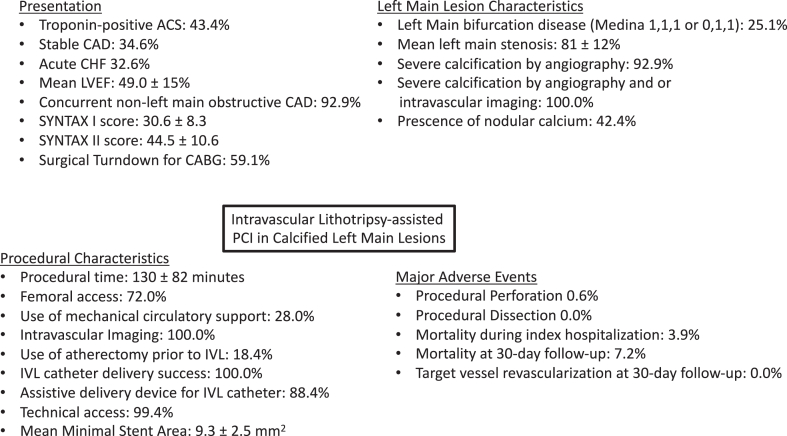
Intravascular lithotripsy-assisted PCI in calcified left main lesions. ACS, acute coronary syndrome; CABG, coronary artery bypass graft; CAD, coronary artery disease; CHF, congestive heart failure; IVL, intravascular lithotripsy; LVEF, left ventricular ejection fraction; SYNTAX, Synergy between PCI with Taxus and Cardiac Surgery.

LMCA PCI is historically associated with increased short- and long-term procedural risk, regardless of clinical presentation.^10^ This risk is heightened in those presenting with ACS.^11^ This all-comers study shows that IVL can be used in this challenging lesion subset with high technical success rates and relatively low risk of complications. Generally, given the myocardial territory subtended by the LMCA coronary distribution, periprocedural MCS is often employed when treating these lesions, particularly in those presenting with ACS.^12^ However, our study showed that the majority (72.0%) of cases did not utilize procedural MCS, despite such a high preponderance of ACS presentations. While formal prospective studies are currently enrolling to help elucidate the role of periprocedural MCS in this patient population, these data suggest that IVL-assisted LMCA PCI in heavily calcified lesions is well tolerated, often without adjunct MCS.^13^

In addition to high technical success rates, there was also a high rate of calcium fracture (82.4%) identified on post-PCI IVI despite the majority (74.3%) of the IVL balloon sizes used being <4 cm in size, which is less than the average minimal luminal area of 4.1 ± 1.3 cm of the LMCA lesions. This supports the premise that IVL effectively modifies calcified plaque in these lesions, even when the vessel diameter exceeds the IVL balloon size, allowing for stent deployment and expansion. However, there was a significant correlation between larger IVL balloon sizes and increased poststent MSA in our study. Given the previously reported improved clinical outcomes correlated with larger MSA during LMCA PCI, this reinforces the current recommendation to use a 1:1-sized IVL balloon based on target vessel diameter.^14^ While the MSA reported in our study is not as large as reported in other smaller series, the final MSA significantly differed across institutions included in our study, likely representing differences in operator technique.^15^

MACE rates were generally low in our cohort, despite complex clinical and anatomic presentations. Final angiographic complications related to the use of IVL were particularly infrequent, consistent with prior reports of IVL use during nonleft main PCI.^6^ There were no reports of vessel dissection and isolated reports of perforation, abrupt vessel closure, slow flow, and no reflow. While rare episodes of most of these events were seen in Disrupt CAD III, they did not report any episodes of slow flow or no reflow. However, our case mix featured a high population of ACS, treatment of bifurcation lesions, and use of concurrent atherectomy. Thus, it is not clear whether these episodes were related to the use of IVL, the complex presentations in which IVL was utilized, and/or rare complications of IVL being seen as use patterns evolve. Thankfully, these complications remain rare, and further study regarding incidence and potential prevention strategies is warranted.

Prior studies evaluating the use of IVL in treating calcified LMCA lesions have been limited to case reports/series, non-IVL specific studies, or were primarily conducted outside the United States (typically Europe, where IVL was approved for use prior to Food and Drug Administration approval in the United States). Rola et al^16^ reported a retrospective series of 16 cases where IVL was used as a second-line therapy for LMCA lesions resistant to vessel preparation with rotational atherectomy and/or non-compliant balloon inflations. They reported 100% technical success with low MACE rates. Cosgrove et al^15^ performed a multicenter retrospective analysis of IVL use in 31 calcified LMCA PCI cases performed at 3 European centers, including reporting of IVI data. Their group also reported excellent technical success rates with low rates of in-hospital MACE.

While our study provides encouraging evidence for the use of IVL-assisted PCI in calcified LMCA lesions, it is important to acknowledge its limitations. The retrospective study design and the lack of a control group limit the conclusions that can be drawn from our findings. Additionally, the short-term follow-up period precludes any assessment of long-term clinical benefits. Future studies with longer follow-up periods are needed to better understand the role of IVL-assisted PCI in managing calcified LMCA disease. Despite these limitations, our study contributes useful data to the growing body of evidence supporting the use of IVL-assisted PCI in calcified coronary lesions, a promising alternative to traditional techniques, especially in patients who are not candidates for surgical revascularization.

## Conclusions

This multicenter retrospective study demonstrated high technical success and low complication rates of IVI-guided, IVL-assisted PCI in patients with calcified LMCA lesions. These findings suggest that IVL-assisted PCI may be a viable treatment option for this challenging patient population. Future studies with long-term follow-up would be useful to expand upon these short-term results.
