# Supplementary material for: Retrospective Multicenter Analysis of Intravascular Lithotripsy Use During Calcified Left Main Coronary Artery Percutaneous Coronary Interventions
Source: J Soc Cardiovasc Angiogr Interv. 2023 Nov 10;3(2):101213. doi: 10.1016/j.jscai.2023.101213 (PMC11307424; doi:10.1016/j.jscai.2023.101213)
Supplement: Supplemental Table 1 [file mmc1.docx]

**Supplemental Table 1. Predictors of 30-day MACE.**

| **Characteristic** | **Odds ratio** | **95% CI** | **P-value** |
| --- | --- | --- | --- |
| **Univariable model** |  |  |  |
| CAD presentation |  |  |  |
| Troponin-positive MI vs. stable | 11.1 | 1.4, 87.8 | 0.02 |
| Unstable angina vs. stable | 5.08 | 0.51, 50.7 | 0.17 |
| Acute CHF, yes vs. no | 3.97 | 1.37, 11.6 | 0.01 |
| CHF, yes vs. no | 4.04 | 1.23, 13.2 | 0.02 |
| Chronic lung disease, yes vs. no | 3.18 | 1.08, 9.36 | 0.04 |
| Chronic kidney disease, yes vs. no | 2.97 | 0.99, 8.93 | 0.05 |
| Surgical turndown, yes vs. no | 4.19 | 0.90, 19.5 | 0.07 |
| SYNTAX II score, per 1-point increase | 1.08 | 0.99, 1.19 | 0.10 |
| Mechanical circulatory support, yes vs. no | 2.17 | 0.76, 6.19 | 0.15 |
| Diabetes mellitus, yes vs. no | 2.11 | 0.73, 6.07 | 0.17 |
| Left main bifurcation lesion, no vs. yes | 1.92 | 0.59, 6.21 | 0.28 |
| Previous MI, yes vs. no | 1.64 | 0.56, 4.80 | 0.37 |
| Minimal stent area, per 1 mm^2^ decrease | 1.11 | 0.88, 1.39 | 0.38 |
| SYNTAX I score, per 1-point increase | 1.04 | 0.94, 1.16 | 0.43 |
| Protected left main disease, no vs. yes | 1.60 | 0.44, 5.88 | 0.48 |
| Atherectomy, no vs. yes | 1.55 | 0.33, 7.25 | 0.58 |
| Sex, female vs. male | 1.15 | 0.37, 3.55 | 0.80 |
| Age, per 5-yr increase | 1.04 | 0.77, 1.39 | 0.83 |
| **Multivariable model** |  |  |  |
| CAD presentation |  |  |  |
| Troponin-positive MI vs. stable | 11.1 | 1.4, 87.8 | 0.02 |
| Unstable angina vs. stable | 5.08 | 0.51, 50.7 | 0.17 |

CAD, coronary artery disease; CHF, congestive heart failure; MACE, major adverse cardiac event; MI, myocardial infarction
